# Assessing the Potential of Algae Extracts for Extending the Shelf Life of Rainbow Trout (*Oncorhynchus mykiss*) Fillets

**DOI:** 10.3390/foods10050910

**Published:** 2021-04-21

**Authors:** María I. Sáez, María D. Suárez, Francisco J. Alarcón, Tomás F. Martínez

**Affiliations:** Departamento de Biología y Geología, Campus de Excelencia Internacional del Mar CEIMAR, Universidad de Almería, 04120 Almería, Spain; msc880@ual.es (M.I.S.); dsuarez@ual.es (M.D.S.); falarcon@ual.es (F.J.A.)

**Keywords:** algae extracts, antioxidants, fish preservatives, total carotenoids, total phenolics, trout fillets

## Abstract

This study evaluates the potential of different algae extracts (*Crassiphycus corneus*, *Cc*; *Ulva ohnoi*, *Uo*; *Arthrospira platensis*, *Ap*; *Haematococcus pluvialis*, *Hp*) as additives for the preservation of rainbow trout fillets. The extracts were prepared with different water to ethanol ratios from the four algae species. The highest ferric reducing antioxidant power (FRAP) was observed in *Uo* extracted in 80% ethanol. *Ap* aqueous extract also had considerable FRAP activity, in agreement with a high total phenolic content. Radical scavenging activity (DPPH) was higher in *Cc* 80% ethanol extract, in agreement with a high total carotenoid content. In fact, when the algae aqueous extracts were assayed on the fish fillets, their antioxidant activity exceeded that of ascorbic acid (ASC). All algae extracts delayed microbial growth and lipid oxidation processes in trout fillets throughout the cold storage period compared to controls, and also improved textural parameters, these effects being more evident for *Ap* and *Hp*. With respect to the color parameters, the *Hp* extract prevented the a* values (redness) from decreasing throughout cold storage, a key point when it comes to colored species, not least salmonids. On the other hand, the *Ap* extract was not as effective as the rest of treatments in avoiding a* and b* decrease throughout the storage period, and thereby the color parameters were impaired. The results obtained, together with the natural origin and the viability for large-scale cultivation, make algae extracts interesting fish preservative agents for the food industry.

## 1. Introduction

The high polyunsaturated fatty acids content in fish fillets contribute to an increased susceptibility to oxidative processes, which leads to decreased shelf life and sensorial quality. Therefore, the prevention of lipid oxidation with natural additives represents a major challenge for the seafood industry [1]. The interest in algae, both macro and microalgae, as a valuable source of potential additives for the food industry has increased considerably in the last few years. They are acknowledged for being rich in a wide variety of bioactive compounds, such as polyphenols and carotenoids, linked to remarkable antioxidant and antimicrobial activities [2]. Thus, some studies have broached the application of algae solutions as natural preservatives for fish products [3,4].

*Crassiphycus* sp. and *Ulva* sp. are two of the most extensively assessed genera of edible macroalgae, owing to their easiness for cultivation, together with their richness in bioactive compounds [5]. On the other hand, the interest in microalgae as natural sources of bioactive compounds is increasing [6], not least taking into consideration that they can be grown at large scale in bioreactors. Their cultivation under controlled conditions not only enables continuous supply, but also guarantees a production free from the eventual bioaccumulation of toxic substances [7]. Among the vast variety of microalgae species, *Haematococcus pluvialis* stands out as a source of astaxanthin [8], a pigment included routinely in commercial feeds for salmonids. With respect to *Arthrospira*, this is one of the most widely assessed cyanobacteria genera in aquaculture, not only owing to its high nutritional value, but also to its acknowledged antioxidant activity [9,10].

In view of the above, this study was aimed at assessing the potential preservative effects of four algae species (*Crassiphycus corneus*, *Ulva ohnoi*, *Arthrospira platensis*, and *Haematococcus pluvialis*) on the shelf life and quality attributes of rainbow trout (*Oncorhynchus mykiss*) fillets kept under cold storage. The phenolic and carotenoid contents, as well as the antioxidant activity of algae extracts prepared with different water to ethanol ratios, were also studied.

## 2. Materials and Methods

### 2.1. Materials

Freeze-dried biomass of two marine macroalgae, *Ulva ohnoi* (*Uo*) and *Crassiphycus corneus* (*Cc*) were provided by LifeBioencapsulation S.L. (Almería, Spain). SABANA project (H2020 EU research program) supplied cyanobacteria *Arthrospira platensis* (*Ap*) and microalgae *Haematococcus pluvialis* (*Hp*) freeze-dried powder. Ascorbic acid was purchased from Sigma-Aldrich (Madrid, Spain).

### 2.2. Preparation of Extracts and Antioxidant Activity

Algal extracts used in antioxidant activity determinations were prepared by mixing 10 g of lyophilized powder with 250 mL ethanol in distilled water at different concentrations (0%, 30%, 50% or 80% *v/v*), according to the procedure described by Santoso et al. [11]. The mixtures were homogenized by vigorous shaking (2 min) and then agitated for 24 h at room temperature (22 °C) in darkness with a magnetic stirrer. Then, the mixtures were centrifuged (8000× *g*, 20 min), and supernatants filtered through Whatman #1 paper. Extractions were carried out in triplicate. Extracts were stored at 4 °C until further use within the next 24 h.

#### 2.2.1. Total Phenolic and Carotenoid Contents

Folin–Ciocalteu spectrophotometric procedure was carried out as described by Singh et al. [12]. A gallic acid standard was prepared (0 to 200 μg mL^−1^) and the results for total phenolic content were expressed as mg of gallic acid equivalents g^−1^. Total carotenoid content of extracts was estimated spectrophotometrically at 470 nm according to the equations proposed by Lichtenthaler and Buschmann [13]. For both parameters, results were expressed as mg g^−1^.

#### 2.2.2. Ferric Reducing Antioxidant Power (FRAP)

The antioxidant capacity of algae extracts was estimated according to the methodology described by Hajimahmoodi et al. [14]. Working solutions were prepared by mixing 100 µL of the samples or standards with 3 mL of FRAP reagent (50 mL of 0.3 M acetate buffer, pH 3.6, 5 mL of 10 mM tripyridyltriazine prepared in 40 mM HCl, and 5 mL of 20 mM FeCl_3_), and kept in the dark for 20 min at room temperature. Absorbance was then measured at 593 nm. Standards were prepared with ethanolic solutions of 6-hydroxy-2,5,7,8-tetramethyl-chroman-2-carboxylic acid (Trolox; Sigma Aldrich), and the results were expressed as μmol Trolox equivalents g^−1^.

#### 2.2.3. Radical Scavenging Activity Determination (DPPH)

This activity was measured according to the method described by Brand-Williams [15]. The reaction mixtures were prepared by adding 75 µL of the algae extracts into 150 µL of 100 μg mL^−1^ 2,2-diphenyl-1-picryl-hydrazyl-hydrate (DPPH) solution, and then incubating at room temperature in the dark for 24 h. The transformation of DPPH from oxidized to reduced form was determined spectrophotometrically at 515 nm. Standards were prepared with ethanolic solutions of Trolox. Results were expressed as μmol Trolox equivalents g^−1^.

### 2.3. Treatment of Fish Fillets with Algae Extracts

#### 2.3.1. Preparation of the Dipping Solutions

Only aqueous algae extracts were used for dipping the fillets. The solutions were prepared by mixing 1.5 g of the respective lyophilized algae with autoclaved distilled water up to a final volume of 500 mL (0.3% *w/v* final concentration). The mixtures were homogenized by vigorous shaking (2 min), then agitated with a magnetic stirrer for 24 h at room temperature (22 °C) in darkness, and filtered through Whatman #1 paper. Ascorbic acid solution (0.3% *w/v*) was also prepared for comparative purposes. The six solutions were designed as follows: (i) control (CONTROL, distilled water); (ii) 0.3% ascorbic acid solution (ASC); (iii) 0.3% *Crassiphycus corneus* extract solution (*Cc*); (iv) 0.3% *Ulva ohnoi* extract solution (*Uo*); (v), 0.3% *Arthrospira platensis* extract solution (*Ap*); and (vi) 0.3% *Haematococcus pluvialis* extract solution (*Hp*). Values of pH and buffering capacity (mEq g^−1^ biomass) of the experimental extracts were determined by acid–base neutralization in order to estimate their possible influence on fillet pH determinations.

#### 2.3.2. Fillet Treatment and Sampling

A total of 60 rainbow trout (500 ± 25 g) were provided by a commercial fish farm (Piscifactorías Andaluzas, Granada, Spain). Immediately after slaughtering, fish were mechanically filleted at the farm, then washed, dried, and kept on ice. Once in the laboratory (less than 2 h after slaughtering), fillets (210 ± 22 g) were distributed in 6 experimental lots of 20 units each (5 sampling points × 4 fillets). The 20 fillets of each treatment group were dipped into the experimental solutions. Autoclaved water was used for dipping control batch. Fillets were carefully placed in sterile trays where the solutions were previously added (500 mL solution per each 4 fillets), kept for 30 s, then removed and placed in a grid for drying during 10 min. Next, fillets were stored in groups of 4 in sterile polyethylene bags, and stored in a cold-room (4 ± 1 °C). Samples were withdrawn from each lot at 1, 4, 6, 8, and 12 days postmortem (dpm) for the experimental determinations.

#### 2.3.3. Microbial Counts

Total viable psychrophilic bacteria counts were carried out according to Sáez et al. [16]. Muscle pieces (1 g) were introduced into aseptic tubes with 10 mL of 0.1% (*w/v*) peptone water (Cultimed SL, Murcia, Spain) and homogenized for 60 s. Four 1-g pieces were withdrawn from each fillet. Appropriate dilutions were serially prepared (12 serial dilutions by mixing 100 µL with 900 µL of peptone water) and then 0.1 mL of each were spread onto plate count agar media. The prepared plates were incubated at 4 °C for 120 h. Microbiological loads were expressed as logarithm of colony-forming units (cfu) g^−1^ tissue.

#### 2.3.4. Determination of Thiobarbituric Acid-Reactive Substances (TBARS)

TBARS were determined according to the method of Buege and Aust [17], as detailed in Molina et al. [18]. Extracts were prepared in triplicate. TBARS value was expressed as mg of malonyldialdehyde (MDA) kg^−1^ fresh tissue.

#### 2.3.5. pH and Water Holding Capacity (WHC)

Fillet pH was determined by means of a penetration electrode as described in [19]. WHC was estimated by weighing 1 cm^3^ samples before and after centrifugation, as described in Suárez et al. [19].

#### 2.3.6. Texture Profile Analysis (TPA)

Texture was measured by compression using a Texture Analyser (TXT2 plus, Stable Micro Systems, Surrey, England, UK), with a load cell of 5 kN. A cylindrical probe (20 mm diameter) was used for pressing downwards into the fillet at a constant speed of 1 mm s^−1^. The determinations were made in all fillets at two adjacent points at the front dorsal muscle. Results are the average of these two values. The probe was placed parallel to the muscle fibers. The parameters hardness, springiness, cohesiveness, gumminess, chewiness, and resilience were calculated as described in Bourne [20].

#### 2.3.7. Fillet Color Determination

Flesh color was measured by L*, a*, and b* system [21], using a Minolta Chroma meter CR400 device (Minolta, Osaka, Japan). The determinations were made at two adjacent points on the front dorsal part of each fillet. The parameters L* (lightness, on a 0–100 point scale from black to white), a* (estimates the position between red, positive values, and green, negative values), and b* (estimates the position between yellow, positive values, and blue, negative values)) were recorded.

### 2.4. Statistics

Determinations on antioxidant activity of extracts were carried out in triplicate, and the effect of the categorical variables “algae species” and “water/ethanol ratio” were determined by analysis of variance (ANOVA). With regard to fillet determinations, the effect of the categorical variables “algae treatment” and “storage time”, as well as their interactions, were determined for each numeric parameter studied by fitting a generalized lineal statistical model (GLM). Least squares means were tested for differences using Fisher’s least significant difference (LSD) procedure. A significance level of 95% was considered to indicate statistical difference. Specific statistical software (SPSS 25, IBM Corporation Inc., Armonk, NY, USA) was used.

## 3. Results and Discussion

### 3.1. Total Polyphenol (TPC) and Carotenoid (TCC) Contents and Antioxidant Activity

The influence of solvent water to ethanol ratio on the characteristics of the algae extracts is shown in Table 1. The *Ap* water extract yielded the highest TPC value among the algae studied, followed by *Hp* and *Cc*, whereas *Uo* showed the lowest value. Previous studies have reported a wide variability in TPC for *Arthrospira* sp., likely as a result of both cultivation factors and extraction processes [22]. With regard to *H. pluvialis*, disparate TPC values have also been reported, attributable to different aspects, such as the culture growth phase and the extraction solvent used [23]. Similar TCP figures were obtained for both seaweeds (*Crassiphycus corneus* and *Ulva ohnoi*), which are within the range reported by other authors [24].

The efficiency of polyphenol extraction depended on the solvent water to ethanol ratio considered (Table 1). Roughly, water extraction was more efficient than the rest of mixtures, in agreement with previous studies [14,23], a fact that might be attributed to the partially polar nature of the phenolic compounds. However, opposite results were reported by Mazumder [22] for *A. platensis*, who observed higher phenolic yield, as well as higher antioxidant activity, after organic solvent extraction, such as 60% ethanol, hexane, or methanol.

With respect to carotenoids (TCC) in microalgae, aqueous extracts yielded higher values for *H. pluvialis* than for *A. platensis*, in line with previous studies [25]. Nevertheless, comparison among different studies should be made cautiously, given that microalgae composition could be influenced by several cultivation factors, mostly nutrient availability and light intensity. With the exception of *Ap*, increasing the proportion of ethanol in the extraction solutions yielded higher TCC in extracts.

With regard to the antioxidant activity of algae extracts, the relative contribution of TPC and TCC to antioxidant capacity has not been well established yet. In our study, FRAP activity was remarkable in *A. platensis* aqueous extracts, in agreement with their high phenolic and carotenoid contents. For seaweed extracts, those with superior TCC values have showed outstanding antioxidant activity (not least DPPH in *Cc* and FRAP in *Uo*). Other authors have reported that phenolics and carotenoids contributed similarly to the antioxidant activity in several microalgae species [23]. The study by Yarnpakdee et al. [26] found a correlation between phenolic content in extracts and FRAP and DPPH values in the seaweed *Cladophora glomerata*. In our study, the aqueous extracts showed a correlation between phenolic contents and FRAP activity (R^2^ = 0.727), as well as between total carotenoid and DPPH activity (R^2^ = 0.990) considering all the algal extracts as a whole. These differences suggest a dissimilar mechanism of action for both groups of compounds.

Since no extensive but only preliminary research has been carried out in this respect in our work, further studies are required to optimize the extraction process of antioxidants for food use, not only in these, but also in many other microalgae species.

Even if the extraction efficiency was higher by using organic solvents in some of the algae species in this study (not least in macroalgae), when it comes to possible practical application, it should be borne in mind that certain organic solvents might not be considered as safe for direct food use, and thus, water extraction might well represent a clear advantage. Moreover, it is reasonable to presume that aqueous extracts would cause a lesser impact on fish fillet sensorial parameters than organic solvent-based extracts. Keeping in mind all the above, only aqueous extracts were considered in the following assays on fillets.

### 3.2. Effects of Extracts on Fillet Quality Parameters

#### 3.2.1. Total Viable Counts

**Figure 1:**
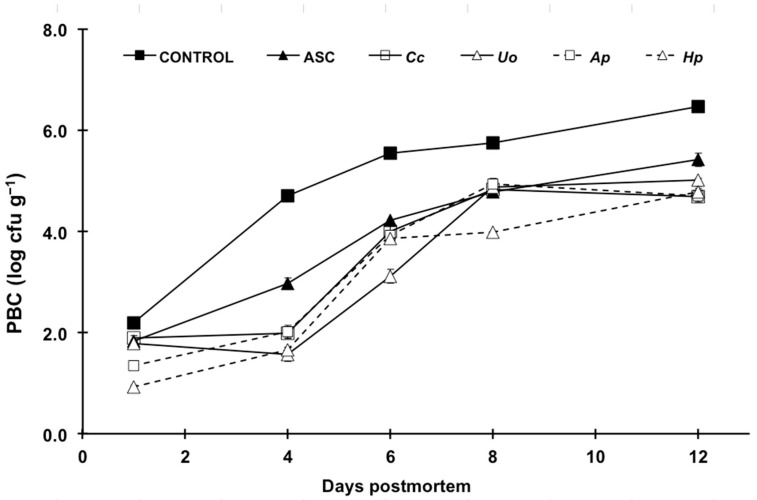
Changes in psychrophilic bacterial counts (PBC) in rainbow trout fillets treated with distilled water (CONTROL); ascorbic acid (ASC) and aqueous extracts of *Crassiphycus corneus* (*Cc*), *Ulva ohnoi* (*Uo*), *Arthrospira platensis* (*Ap*) and *Haematococcus pluvialis* (*Hp*) during a 12-day cold storage (4 °C) period. Values are expressed as mean ± sd. CFU stands for colony-forming units.

Initial psychrophilic bacterial count in control fillets was 2.2 log CFU g^−1^, and increased over storage time, exceeding the maximum acceptable limit for fish (6 log CFU g^−1^; ICMSF [27]) at 12 dpm. Compared to controls, all treatments inhibited bacterial growth in fillets to a greater or lesser degree (Figure 1, Table S1). ASC markedly delayed bacterial growth in trout fillets, in agreement with previous studies [28,29]. Indeed, this compound with acknowledged antimicrobial activity is, up until now, one of the very few additives authorized for unprocessed fresh fish in the EU (Regulation 1333/2008/EC) [30].

In this regard, it is remarkable that the inhibition of microbial growth caused by all the aqueous algae extracts was significantly more intense than that observed for ASC, not least during the first six days of the storage period. The effects of the *H. pluvialis* and *A. platensis* solutions outweighed the other extracts at the initial stages of cold storage. These results agree with the antimicrobial efficiency reported for the seaweed *Fucus spiralis* on refrigerated hake [31].

#### 3.2.2. Lipid Oxidation (TBARS)

**Figure 2:**
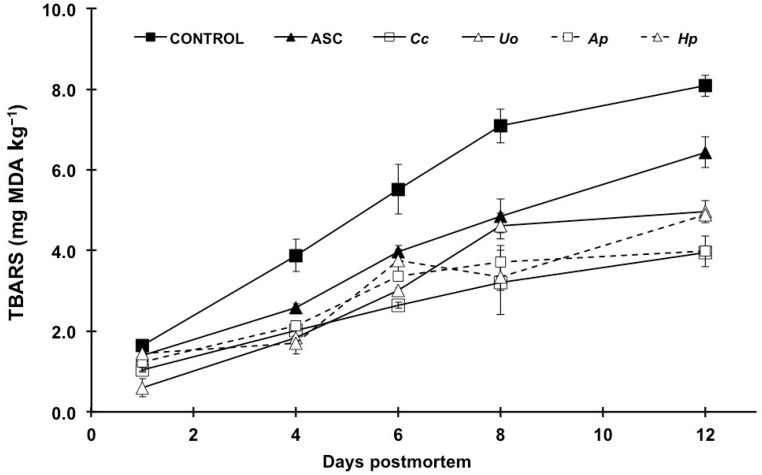
Time course of lipid oxidation in rainbow trout fillets treated with distilled water (CONTROL); ascorbic acid (ASC) and aqueous extracts of *Crassiphycus corneus* (*Cc*), *Ulva ohnoi* (*Uo*), *Arthrospira platensis* (*Ap*) and *Haematococcus pluvialis* (*Hp*) during a 12-day cold storage (4 °C) period. Values are given as mean ± sd. TBARS stands for thiobarbituric acid reactive substances, expressed as mg malonyldialdehyde (MDA) kg^−1^.

The initial TBARS content (Figure 2) in fillets was within the values established for good quality fish products (1–2 mg MDA kg^−1^) [32]. The values increased during storage in all treatments, but ASC showed remarkably lower values than controls, confirming its powerful antioxidant effects [29,33]. Interestingly, the seaweed extracts assessed also displayed outstanding effectiveness in protecting muscle lipids from oxidative processes (Figure 2, Table S2), which is the same as described in previous studies on different fish products (*Durvillaea antartica*, *Ulva lactuca*, and *Pyropia columbina* on canned salmon, [3]; *Fucus spiralis* on fresh hake, [30]; *Cladophora glomerata* on sliced tuna, [26]). Moreover, it should be pointed out that the algae extracts not only exceeded the antioxidant capacity of ASC, but also the effects persisted longer than those of ASC up to the end of the storage period.

Surprisingly, despite their potent antioxidant activity, research on the use of microalgae with the purpose of preserving fish products is not extensive. Takyar et al. [34] found that ethanolic extracts of *Chlorella vulgaris* and *A. platensis* yielded significant antioxidant activity on rainbow trout fillets. Studies on other food products also reported remarkable antioxidant effects (*A. platensis* in olive oil, [35]; *H. pluvialis* in ground pork [36]).

#### 3.2.3. pH and Water Holding Capacity (WHC)

**Figure 3:**
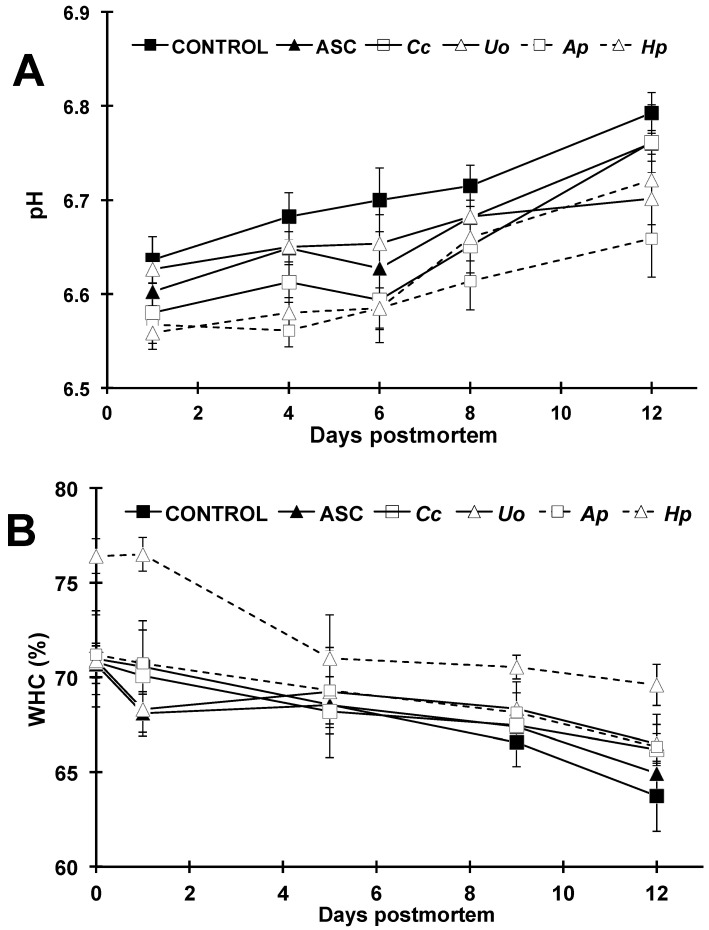
Postmortem changes in pH (**A**) and water holding capacity (WHC; **B**) of rainbow trout fillets treated with distilled water (CONTROL); ascorbic acid (ASC) and aqueous extracts of *Crassiphycus corneus* (*Cc*), *Ulva ohnoi* (*Uo*), *Arthrospira platensis* (*Ap*) and *Haematococcus pluvialis* (*Hp*) during a 12-day cold storage (4 °C) period. Values are expressed as mean ± sd.

The postmortem changes in pH and WHC are shown in Figure 3 and Table S3. The values increased significantly throughout the 12-day trial for pH in all experimental batches, likely owing to the emergence of alkaline compounds from protein bacterial degradation [37]. The significantly lower pH values observed in all treatments compared to the control fillets (being the lowest those caused by *Ap* and *Hp* extracts) might well be attributed to the antimicrobial effects also observed (Figure 1), which is same as found in previous studies [29]. Nevertheless, doubts can arise regarding the possible influence on fillet pH of the dipping solutions themselves, and therefore, this was also assessed. Overall, the extracts prepared at the concentration assayed (3 g L^−1^) yielded pH values within half a pH unit from neutrality (Table 2), except *H. pluvialis* and, especially, ASC, which showed a clearly acidic pH. Not only the pH value, but also the buffering capacity was estimated, and the results indicated that, with the exception of ASC, this parameter can be considered as negligible.

No evidence pointing to any effect of dipping solutions on fillet pH was observed, not even for ASC, likely owing to the fact that pH was not measured on fillet surface, but in dorsal muscle depth by means of a penetration electrode.

With regard to WHC, this parameter decreased throughout storage time, with the remaining values being statistically higher for *Hp*-treated fillets during the entire trial compared to the rest of treatments. This fact could indicate significant contribution of this extract in maintaining muscle mechanical properties, which is in agreement with the improvement observed in the textural parameters as well.

#### 3.2.4. Texture Profile Analysis (TPA)

The TPA parameters are shown in Table 3. The hardness decreased in all treatments during cold storage, although all treatments yielded consistently higher values for this parameter than the control fillets at any sampling time from day one onwards. The postmortem deterioration of the textural parameters (not least muscle softening) is caused by myofibrillar and connective tissue proteolysis, which leads to a relaxation of the muscle structure [38]. Not a single factor, but a complex constellation of them (both biochemical and microbiological) is responsible for the alterations of the muscle structures throughout storage time, this ending in the unacceptable softening of the fresh fish. Given that muscle hardness is crucial in terms of purchasing decision, any strategy aimed at preserving this parameter deserves attention.

The delayed softening in all treated fillets might well have been linked to lower microbial counts (Figure 1), which is also in agreement with a recent study on rainbow trout fillets [29]. In addition, the inhibition of muscle endogenous protease activity owing to the antioxidant effect of the additives might also have occurred [39]. Although all the algae treatments kept a firmness above that of control fillets, it is remarkable that *Ap* and *Hp* were especially effective in this regard, outweighing even the effects of ASC from day six onwards. This might be related to the higher antioxidant activity observed for those aqueous extracts (Table 1). On the other hand, no consistent trend was observed for the rest of the texture parameters measured (gumminess, chewiness, cohesiveness and resilience) neither regarding storage time nor additive treatment.

#### 3.2.5. Instrumental Color

The influence of the experimental treatments on fillet color parameters is shown in Table 4. The lightness (L*) values increased over time in all treatments. Such an increase has been attributed in salmonids to lipid oxidation and protein denaturation, both effects together leading to higher light refraction on the fillet surface [40]. Color-related quality loss in trout fillets during cold storage results from the combination of increased L*, and decreased a* and b* parameters, together jeopardizing their market value [41]. Such loss of pigmentation has also been partly attributed to astaxanthin degradation [42], included routinely in finishing diets for farmed salmonids, and responsible for their distinctive color.

The most evident effect of algae extracts on L* was the significant decrease in this parameter up to 12 dpm caused by *Ap*, compared to the controls and to the rest of the lots, which, roughly, were similar. The *Ap* extract also caused a clear detrimental effect on the a* and b* parameters (as also found by Takyar et al. [34]). *Uo* also decreased fillet redness throughout the complete storage period, and it is likely that this effect can be attributed to the richness of *Uo* and *Ap* in chlorophylls and phycocyanins, respectively.

At the other end, the *Hp* extract accounted not only for yielding the highest a* and b* values among treatments, but also for causing the most persistent effects during the entire storage period (Table 4), a very desirable effect from the point of view of market acceptability. It is likely that the outstanding astaxanthin content in *Hp* was responsible not only for these favorable effects on color properties, but also for the remarkably positive antimicrobial, antioxidant, and textural effects found in this work, which makes this microalga a promising candidate as a fish preservative in colored muscle fillets.

However, when it comes to practical utilization, there is a clear incompatibility between the intense antioxidant and antimicrobial activities of *Uo* and *Ap* extracts and their coloring properties, which could limit their applicability on fresh fillets of white muscle fish species.

## 4. Conclusions

The results indicate that all the algae species tested showed valuable antioxidant and antimicrobial effects, which might well be linked to their richness in both carotenoid and phenolic compounds. Outstanding FRAP and DPPH activities were found in *A. platensis* aqueous extract, in agreement with the highest phenolic content. Furthermore, the seaweed extracts with superior carotenoid contents yielded the highest antioxidant capacity (*C. corneus* and *U. ohnoi*).

Not only the antioxidant, but also the antimicrobial effects of the aqueous extracts of all the algae species tested were noteworthy, outweighing even those caused by ascorbic acid, the most widely used authorized additive for fresh fish. Together with delayed firmness loss, all the algae extracts assayed were able to extend the shelf life of the trout fillets compared to untreated controls.

When the impact on trout fillet color is also included in the overall assessment, *H. pluvialis* exceeded the effects of the other species, given that this extract lacks the detrimental effects observed for *A. platensis* on color parameters, despite its powerful antioxidant and antimicrobial activity. In view of the results, extracts of the algae species tested could represent valuable alternative sources of natural food additives with remarkable effects on fresh fish objective quality parameters. In addition to favorable antioxidant and antimicrobial properties, the extracts did not cause negative impact on trout flesh color parameters, which are considered crucial quality attributes for this species.

## Figures and Tables

**Table 1:** Total phenolic and carotenoid contents and antioxidant activity of algae extracts prepared with different water to ethanol ratios.

Algae Species	W/EtOH	TPC	TCC	FRAP	DPPH
*Crassiphycus corneus*	100/0	1.38 ± 0.01 ^B,b^	1.63 ± 0.08 ^B,a^	3.90 ± 0.49 ^A,a^	9.4 ± 0.61 ^B,a^
	70/30	0.98 ± 0.03 ^A,a^	2.08 ± 001 ^B,b^	3.51 ± 0.06 ^A,a^	21.55 ± 0.05 ^C,c^
	50/50	0.97 ± 0.03 ^A,a^	2.49 ± 0.03 ^C,c^	4.34 ± 0.42 ^A,b^	19.02 ± 0.71 ^C,b^
	20/80	1.12 ± 0.02 ^A,b^	3.46 ± 0.08 ^D,d^	4.14 ± 0.29 ^A,b^	23.5 ± 0.9 ^D,d^
*Ulva ohnoi*	100/0	1.18 ± 0.18 ^A,b^	1.16 ± 0.09 ^A,a^	8.37 ± 0.06 ^C,a^	4.14 ± 0.16 ^A,c^
	70/30	1.08 ± 0.02 ^B,a^	1.41 ± 0.09 ^A,b^	8.83 ± 0.13 ^B,b^	4.56 ± 0.34 ^A,c^
	50/50	1.08 ± 0.03 ^B,a^	1.68 ± 0.06 ^B,c^	8.62 ± 0.06 ^C,b^	3.56 ± 0.30 ^A,b^
	20/80	1.42 ± 0.15 ^B,b^	1.86 ± 0.15 ^B,c^	16.52 ± 0.18 ^C,c^	3.05 ± 0.08 ^A,a^
*Artrhospira platensis*	100/0	3.22 ± 0.07 ^D,c^	1.62 ± 0.36 ^B^	13.93 ± 0.02 ^D,b^	9.82 ± 0.40 ^B,c^
	70/30	1.85 ± 0.02 ^C,b^	1.32 ± 0.03 ^A^	13.13 ± 0.01 ^C,b^	8.43 ± 0.29 ^B,b^
	50/50	1.37 ± 0.01 ^C,a^	1.50 ± 0.01 ^A^	6.13 ± 0.66 ^B,a^	5.37 ± 0.05 ^B,a^
	20/80	1.40 ± 0.02 ^B,a^	1.57 ± 0.04 ^A^	6.55 ± 0.61 ^B,a^	5.16 ± 0.04 ^B,a^
*Haematococcus pluvialis*	100/0	1.64 ± 0.03 ^C^	2.06 ± 0.02 ^C,a^	6.40 ± 0.18 ^B,a^	13.66 ± 0.15 ^C,a^
	70/30	1.62 ± 0.02 ^C^	2.23 ± 0.21 ^C,b^	6.53 ± 0.22 ^B^	13.58 ± 0.17 ^C^
	50/50	1.67 ± 0.01 ^C^	2.44 ± 0.15 ^C,c^	6.64 ± 0.34 ^B^	13.22 ± 0.31 ^C^
	20/80	1.69 ± 0.07 ^C^	2.52 ± 0.03 ^C,b^	6.65 ± 0.22 ^B,b^	14.61 ± 0.11 ^C,b^

W/EtOH: water to ethanol ratio in solvents. TPC: total phenolic content of extracts, expressed as mg of gallic acid equivalents per g. TCC: total carotenoid content of extracts, expressed as μmol equivalents Trolox per g. FRAP: ferric reducing antioxidant power, expressed as μmol equivalent of Trolox per g. DPPH: radical scavenging activity, expressed as μmol equivalents Trolox per g. Superscript uppercase letters indicate differences attributable to algae species for the same water/ethanol proportion. Superscript lowercase letters indicate differences attributable to water/ethanol proportion for the same algal species. Values are mean ± sd on dry weight basis.

**Table 2:** Values of pH and buffering capacity measured for the aqueous dipping solutions (0.3% *w/v*).

Dipping Solutions	pH	Buffering Capacity (mEq L^−1^) *
ASC	3.14 ± 0.03	14.67 ± 0.08
*Ulva ohnoi*	6.93 ± 0.02	0.03 ± 0.01
*Crassiphycus corneus*	6.64 ± 0.02	0.12 ± 0.02
*Artrhospira platensis*	4.62 ± 0.01	0.47 ± 0.03
*Haematococcus pluvialis*	7.12 ± 0.03	0.31 ± 0.05

* Acidic extracts (ASC, *U. ohnoi*, *C. corneus*, *H. pluvialis*) were neutralized with 0.1 N NaOH, whereas alkaline extracts (*A. platensis*) were neutralized with 0.1 N HCl.

**Table 3:** Changes in texture profile analysis parameters of rainbow trout fillets treated with algae aqueous extracts throughout a 12-day cold storage (4 °C) period.

	dpm	C	ASC	*Cc*	*Uo*	*Ap*	*Hp*	*p*
Hardness (N)	1	18.6 ± 1.2 ^B^	19.9 ± 1.4 ^B^	18.7 ± 0.6	19.8 ± 2.4 ^B^	19.8 ± 1.2 ^B^	20.6 ± 1.3 ^B^	0.133
	4	16.4 ± 1.2 ^B,a^	19.8 ± 3.3 ^B,ab^	17.6 ± 2.1 ^ab^	19.4 ± 1.4 ^Bab^	21.1 ± 1.2 ^Bb^	22.1 ± 1.3 ^B,b^	0.004
	6	16.7 ± 1.5 ^B,a^	18.2 ± 1.5 ^AB,a^	18.1 ± 1.5 ^a^	18.7 ± 1.2 ^BC,a^	20.9 ± 1.7 ^B,b^	22.2 ± 1.7 ^B,b^	<0.001
	8	16.6 ± 1.4 ^B,a^	17.4 ± 1.7 ^AB,a^	17.4 ± 1.6 ^B,a^	18.2 ± 1.3 ^B,b^	17.2 ± 1.5 ^B,b^	19.0 ± 1.8 ^B,c^	0.005
	12	13.7 ± 1.9 ^A,a^	16.4 ± 1.2 ^A,b^	16.2 ± 1.6 ^b^	14.8 ± 1.2 ^A,ab^	16.4 ± 1.9 ^A,b^	15.9 ± 2.1 ^A,b^	0.007
	*p*	0.001	0.001	0.061	<0.001	0.002	<0.001	
Springiness (mm)	1	0.72 ± 0.05 ^A^	0.76 ± 0.04	0.75 ± 0.07 ^A^	0.80 ± 0.07 ^A^	0.73 ± 0.05 ^A^	0.78 ± 0.06 ^AB^	0.097
	4	0.76 ± 0.05 ^A,a^	0.78 ± 0.04 ^a^	0.89 ± 0.05 ^B,b^	0.85 ± 0.05 ^B,b^	0.90 ± 0.05 ^C,b^	0.91 ± 0.03 ^C,b^	<0.001
	6	0.82 ± 0.04 ^B,a^	0.80 ± 0.07 ^a^	0.84 ± 0.06 ^B,b^	0.79 ± 0.05 ^A,a^	0.89 ± 0.07 ^C,c^	0.87 ± 0.05 ^C,c^	0.003
	8	0.76 ± 0.06 ^A,a^	0.74 ± 0.06 ^a^	0.76 ± 0.04 ^A,a^	0.78 ± 0.03 ^A,ab^	0.83 ± 0.05 ^B,b^	0.81 ± 0.02 ^B,b^	0.006
	12	0.73 ± 0.09 ^A,a^	0.74 ± 0.08 ^ab^	0.75 ± 0.06 ^A,b^	0.74 ± 0.08 ^A,ab^	0.76 ± 0.05 ^A,b^	0.76 ± 0.05 ^A,b^	0.018
	*p*	*0.020*	*0.099*	*<0.001*	*0.011*	*<0.001*	*<0.001*	
Cohesiveness	1	0.16 ± 0.03 ^b^	0.16 ± 0.01 ^b^	0.13 ± 0.03 ^A,a^	0.17 ± 0.03 ^B,b^	0.15 ± 0.03 ^b^	0.16 ± 0.03 ^b^	0.010
	4	0.15 ± 0.02 ^a^	0.15 ± 0.02 ^a^	0.16 ± 0.02 ^C^	0.14 ± 0.01 ^Aa^	0.17 ± 0.02 ^b^	0.17 ± 0.02 ^b^	0.018
	6	0.14 ± 0.01 ^a^	0.15 ± 0.01 ^a^	0.16 ± 0.02 ^C,b^	0.13 ± 0.01 ^Aa^	0.17 ± 0.01 ^bc^	0.18 ± 0.03 ^c^	0.009
	8	0.15 ± 0.01	0.15 ± 0.03	0.14 ± 0.02 ^B^	0.17 ± 0.02 ^B^	0.15 ± 0.01	0.15 ± 0.03	0.932
	12	0.16 ± 0.02	0.16 ± 0.03	0.14 ± 0.02 ^B^	0.15 ± 0.03 ^AB^	0.15 ± 0.03	0.15 ± 0.02	0.557
	*p*	0.083	0.511	0.012	0.016	0.063	0.107	
Gumminess (N mm^−2^)	1	2.8 ± 0.2 ^C^	2.7 ± 0.3	2.7 ± 0.3 ^B^	2.7 ± 0.4 ^B^	2.9 ± 0.3 ^B^	2.9 ± 0.3 ^B^	0.538
	4	2.5 ± 0.3 ^BC,a^	3.1 ± 0.7 ^ab^	3.5 ± 0.4 ^D,b^	2.7 ± 0.3 ^B,ab^	3.5 ± 0.5 ^C,b^	3.3 ± 0.4 ^C,ab^	0.005
	6	2.5 ± 0.6 ^BC,a^	2.6 ± 0.4 ^a^	3.0 ± 0.5 ^C,c^	2.8 ± 0.3 ^BC,b^	3.1 ± 0.4 ^B,c^	3.3 ± 0.4 ^C,c^	0.004
	8	2.3 ± 0.2 ^A,a^	2.6 ± 0.3 ^b^	2.2 ± 0.4 ^Aa^	3.1 ± 0.3 ^C,c^	2.8 ± 0.2 ^AB,b^	2.8 ± 0.2 ^B,b^	0.001
	12	2.2 ± 0.2 ^A,a^	2.6 ± 0.6 ^c^	2.4 ± 0.4 ^AB,bc^	1.9 ± 0.3 ^A,a^	2.5 ± 0.4 ^A,c^	2.2 ± 0.4 ^A^	0.004
	*p*	0.022	0.261	<0.001	<0.001	0.001	<0.001	
Chewiness (N mm^−1^)	1	1.9 ± 0.3 ^a^	2.2 ± 0.5 ^BC,ab^	2.2 ± 0.4 ^AB,ab^	2.6 ± 0.4 ^BC,b^	2.3 ± 0.3 ^AB^	2.3 ± 0.3 ^B^	0.004
	4	2.1 ± 0.4 ^a^	2.5 ± 0.3 ^D,ab^	2.8 ± 0.4 ^B,c^	2.3 ± 0.3 ^B,ab^	3.1 ± 0.4 ^B,c^	2.7 ± 0.3 ^BC,bc^	0.003
	6	1.9 ± 0.5 ^a^	2.3 ± 0.4 ^BC,ab^	2.7 ± 0.4 ^B,b^	2.1 ± 0.4 ^B,a^	2.7 ± 0.4 ^B,b^	3.3 ± 0.3 ^C,c^	<0.001
	8	1.9 ± 0.3 ^a^	1.8 ± 0.4 ^A,a^	1.8 ± 0.2 ^A^	2.5 ± 0.4 ^BC,b^	1.8 ± 0.1 ^A,a^	2.3 ± 0.4 ^B,b^	0.010
	12	1.6 ± 0.4 ^b^	1.9 ± 0.5 ^AB,c^	1.9 ± 0.6 ^A,c^	1.3 ± 0.4 ^A,a^	1.9 ± 0.3 ^A,c^	1.6 ± 0.3 ^Ab^	0.001
	*p*	0.149	0.008	<0.001	<0.001	<0.001	<0.001	
Resilience (N mm^−1^)	1	0.16 ± 0.03 ^b^	0.17 ± 0.01 ^c^	0.13 ± 0.03 ^A,a^	0.17 ± 0.03 ^B,c^	0.15 ± 0.03 ^ab^	0.16 ± 0.03 ^b^	0.021
	4	0.15 ± 0.02	0.15 ± 0.03	0.16 ± 0.02 ^B,b^	0.14 ± 0.01 ^A^	0.17 ± 0.02	0.17 ± 0.02	0.118
	6	0.14 ± 0.01 ^a^	0.16 ± 0.02 ^b^	0.16 ± 0.02 ^A,b^	0.13 ± 0.01 ^A,a^	0.16 ± 0.02 ^b^	0.18 ± 0.03 ^c^	0.009
	8	0.15 ± 0.01	0.15 ± 0.03	0.14 ± 0.02 ^A^	0.15 ± 0.02 ^A^	0.15 ± 0.01	0.15 ± 0.02	0.932
	12	0.16 ± 0.02	0.16 ± 0.03	0.14 ± 0.02 ^A^	0.15 ± 0.03 ^A^	0.15 ± 0.03	0.15 ± 0.03	0.557
	*p*	0.083	0.511	<0.001	0.017	0.063	0.107	

dpm: days postmortem. C: control (distilled water). ASC: ascorbic acid. *Cc*: *Crassiphycus corneus*. *Uo*: *Ulva ohnoi*. *Ap*: *Arthrospira platensis*. *Hp*: *Haematococcus pluvialis*. Values are mean ± sd. Superscript uppercase letters indicate differences (*p* < 0.05) attributable to storage time within each additive treatment. Superscript lowercase letters indicate differences (*p* < 0.05) attributable to treatments within each storage time.

**Table 4:** Changes in color parameters of rainbow trout fillets treated with algae aqueous extracts throughout a 12-day cold storage (4 °C) period.

	dpm	C	ASC	*Cc*	*Uo*	*Ap*	*Hp*	*p*
L*	1	45.7 ± 1.3 ^A,b^	45.7 ± 0.7 ^A,b^	44.5 ± 0.7 ^A,b^	46.7 ± 0.9 ^AB,c^	43.3 ± 0.8 ^A,a^	45.1 ± 0.5 ^A,b^	0.012
	4	45.5 ± 1.2 ^A,b^	48.7 ± 1.2 ^B,c^	45.5 ± 0.9 ^A,b^	45.0 ± 0.6 ^A,b^	43.0 ± 0.8 ^A,a^	46.1 ± 0.9 ^AB,b^	<0.001
	8	47.8 ± 1.4 ^B,b^	49.0 ± 0.7 ^B,b^	48.9 ± 0.8 ^B,b^	48.3 ± 0.7 ^B,b^	45.2 ± 0.7 ^B,a^	47.3 ± 1.1 ^B,b^	<0.001
	12	48.4 ± 1.5 ^B,a^	51.8 ± 1.2 ^C,bc^	52.8 ± 0.9 ^C,c^	48.5 ± 0.9 ^C,a^	48.1 ± 0.7 ^C,a^	50.3 ± 1.3 ^C,b^	0.004
	*p*	0.001	<0.001	<0.001	0.001	<0.001	<0.001	
a*	1	12.2 ± 0.4 ^B,c^	9.7 ± 0.4 ^A,b^	11.9 ± 0.6 ^c^	9.9 ± 0.4 ^B,b^	8.7 ± 0.4 ^B,a^	12.4 ± 0.5 ^c^	<0.001
	4	12.5 ± 0.1 ^B,c^	11.1 ± 0.4 ^B,b^	11.7 ± 0.2 ^bc^	10.5 ± 0.3 ^B,bc^	9.1 ± 0.2 ^B,a^	12.1 ± 0.4 ^bc^	<0.001
	8	11.5 ± 0.3 ^AB,d^	11.2 ± 0.5 ^B,d^	10.9 ± 0.3 ^c^	8.5 ± 0.3 ^A,b^	6.3 ± 0.8 ^A,a^	12.5 ± 0.4 ^e^	<0.001
	12	10.3 ± 0.3 ^A,c^	10.9 ± 0.3 ^B,d^	10.3 ± 0.3 ^d^	9.4 ± 0.4 ^A,b^	6.7 ± 0.1 ^A,a^	12.5 ± 0.4 ^e^	<0.001
	*p*	0.001	0.002	0.098	<0.001	0.001	0.143	
b*	1	13.2 ± 0.2 ^B,c^	9.6 ± 0.4 ^A,a^	11.5 ± 0.4 ^B,b^	11.4 ± 0.4 ^AB,b^	10.3 ± 0.6 ^a^	13.5 ± 0.9 ^AB,c^	0.001
	4	12.6 ± 0.7 ^AB,b^	11.9 ± 1.1 ^B,b^	10.8 ± 1.1 ^A,ab^	12.0 ± 1.2 ^AB,b^	9.8 ± 1.2 ^a^	13.1 ± 1.0 ^A,b^	0.013
	8	11.4 ± 0.5 ^A,b^	11.4 ± 1.1 ^AB,b^	10.2 ± 0.7 ^A,ab^	10.7 ± 0.7 ^A,ab^	9.5 ± 0.6 ^a^	13.7 ± 0.4 ^AB,c^	0.008
	12	11.0 ± 0.4 ^A,ab^	13.0 ± 0.7 ^B,bc^	11.6 ± 0.8 ^B,ab^	12.8 ± 0.8 ^B,bc^	9.0 ± 0.9 ^a^	14.1 ± 0.5 ^B,c^	<0.001
	*p*	0.024	0.006	<0.001	0.011	0.376	<0.001	

dpm: days postmortem. C: control (distilled water). ASC: ascorbic acid. *Cc*: *Crassiphycus corneus*. *Uo*: *Ulva ohnoi*. *Ap*: *Arthrospira platensis*. *Hp*: *Haematococcus pluvialis*. Values are mean ± sd. Superscript uppercase letters indicate differences (*p* < 0.05) attributable to storage time within each additive treatment. Superscript lowercase letters indicate differences (*p* < 0.05) attributable to treatments within each storage time.

## Data Availability

Data available on request.

## Supplementary Materials

The following are available online at https://www.mdpi.com/article/10.3390/foods10050910/s1, Table S1: Changes in psychrophilic bacterial counts (PBC, log cfu g^−1^) in rainbow trout fillets treated with distilled water (C); ascorbic acid (ASC) and aqueous extracts of *Crassiphycus corneus* (*Cc*), *Ulva ohnoi* (*Uo*), *Arthrospira platensis* (*Ap*) and *Haematococcus pluvialis* (*Hp*) during a 12-day cold storage (4 °C) period. Values are given as mean ± standard deviation (n = 4 fillets), Table S2: Changes in lipid oxidation (estimated as mg MDA kg^−1^ content) in rainbow trout fillets treated with distilled water (C); ascorbic acid (ASC) and aqueous extracts of *Crassiphycus corneus* (*Cc*), *Ulva ohnoi* (*Uo*), *Arthrospira platensis* (*Ap*) and *Haematococcus pluvialis* (*Hp*) during a 12-day cold storage (4 °C) period. Values are given as mean ± standard deviation (n = 4 fillets), Table S3: Changes in pH and water holding capacity (WHC) in rainbow trout fillets treated with distilled water (C); ascorbic acid (ASC) and aqueous extracts of *Crassiphycus corneus* (*Cc*), *Ulva ohnoi* (*Uo*), *Arthrospira platensis* (*Ap*) and *Haematococcus pluvialis* (*Hp*) during a 12-day cold storage (4 °C) period. Values are given as mean ± standard deviation (n = 4 fillets).
