# Supplementary material for: Assessing the Potential of Algae Extracts for Extending the Shelf Life of Rainbow Trout (Oncorhynchus mykiss) Fillets
Source: Foods. 2021 Apr 21;10(5):910. doi: 10.3390/foods10050910 (PMC8143106; doi:10.3390/foods10050910)
Supplement: Supplementary file 1 [file foods-10-00910-s001.zip › foods-1139294-SI.pdf]

Table S1. Changes in psychrophilic bacterial counts (PBC, log cfu g^{-1}) in rainbow trout fillets treated with distilled water (C); ascorbic acid (ASC) and aqueous extracts of *Crassiphycus corneus* (*Cc*), *Ulva ohnoi* (*Uo*), *Arthrospira platensis* (*Ap*) and *Haematococcus pluvialis* (*Hp*) during a 12-day cold storage (4 °C) period. Values are given as mean ± standard deviation (n = 4 fillets).

dpm	C	ASC	*Cc*	*Uo*	*Ap*	*Hp*	*p*
1	2.2 ± 0.1 ^{A,d}	1.8 ± 0.1 ^{A,c}	1.9 ± 0.1 ^{A,c}	1.8 ± 0.1 ^{A,c}	1.3 ± 0.1 ^{A,b}	0.9 ± 0.1 ^{A,a}	<0.001
4	4.7 ± 0.1 ^{B,d}	3.0 ± 0.1 ^{B,c}	2.0 ± 0.1 ^{A,b}	1.6 ± 0.1 ^{A,a}	2.0 ± 0.1 ^{B,b}	1.7 ± 0.1 ^{B,a}	<0.001
6	5.5 ± 0.2 ^{C,d}	4.2 ± 0.1 ^{C,c}	4.0 ± 0.1 ^{B,bc}	3.1 ± 0.1 ^{B,a}	3.9 ± 0.1 ^{C,b}	3.9 ± 0.1 ^{C,b}	0.001
8	5.7 ± 0.1 ^{C,c}	4.8 ± 0.1 ^{D,b}	4.8 ± 0.1 ^{C,b}	4.9 ± 0.1 ^{C,b}	4.9 ± 0.1 ^{D,b}	5.0 ± 0.3 ^{D,a}	<0.001
12	6.5 ± 0.1 ^{D,c}	5.4 ± 0.1 ^{E,a}	4.7 ± 0.2 ^{C,a}	5.0 ± 0.1 ^{C,a}	4.7 ± 0.1 ^{D,a}	4.8 ± 0.1 ^{D,a}	0.001
p	<0.001	<0.001	<0.001	<0.001	<0.001	<0.001	

Superscript uppercase letters indicate differences attributable to storage time within each treatment. Superscript lowercase letters indicate differences attributable to treatments within each storage time ($p < 0.05$). dpm: days postmortem.

Table S2. Changes in lipid oxidation (estimated as mg MDA kg^{-1} content) in rainbow trout fillets treated with distilled water (C); ascorbic acid (ASC) and aqueous extracts of *Crassiphycus corneus* (*Cc*), *Ulva ohnoi* (*Uo*), *Arthrospira platensis* (*Ap*) and *Haematococcus pluvialis* (*Hp*) during a 12-day cold storage (4 °C) period. Values are given as mean ± standard deviation (n = 4 fillets).

dpm	C	ASC	*Cc*	*Uo*	*Ap*	*Hp*	*p*
1	1.6 ± 0.1 ^{A,d}	1.4 ± 0.2 ^{A,c}	1.0 ± 0.1 ^{A,b}	0.6 ± 0.2 ^{A,a}	1.2 ± 0.1 ^{A,bc}	1.4 ± 0.1 ^{A,c}	0.023
4	3.9 ± 0.4 ^{B,d}	2.6 ± 0.1 ^{B,c}	2.0 ± 0.2 ^{B,b}	1.8 ± 0.4 ^{B,ab}	2.1 ± 0.1 ^{B,b}	1.7 ± 0.1 ^{A,a}	<0.001
6	5.5 ± 0.6 ^{C,e}	3.9 ± 0.1 ^{C,d}	2.6 ± 0.3 ^{BC,a}	3.0 ± 0.1 ^{C,b}	3.4 ± 0.1 ^{C,c}	3.8 ± 0.2 ^{B,d}	<0.001
8	7.1 ± 0.4 ^{D,c}	4.9 ± 0.4 ^{D,b}	3.2 ± 0.3 ^{B,a}	4.6 ± 0.3 ^{D,b}	3.7 ± 0.4 ^{CD,a}	3.4 ± 0.4 ^{B,a}	0.001
12	8.1 ± 0.3 ^{D,d}	6.4 ± 0.4 ^{E,c}	3.9 ± 0.2 ^{C,a}	4.0 ± 0.3 ^{D,a}	3.9 ± 0.1 ^{D,a}	4.9 ± 0.1 ^{C,b}	<0.001
p	<0.001	<0.001	0.001	<0.001	<0.001	<0.001	

Superscript uppercase letters indicate differences attributable to storage time within each treatment. Superscript lowercase letters indicate differences attributable to treatments within each storage time ($p < 0.05$). dpm: days postmortem.

Table S3. Changes in pH and water holding capacity (WHC) in rainbow trout fillets treated with distilled water (C); ascorbic acid (ASC) and aqueous extracts of *Crassiphycus corneus* (*Cc*), *Ulva ohnoi* (*Uo*), *Arthrospira platensis* (*Ap*) and *Haematococcus pluvialis* (*Hp*) during a 12-day cold storage (4 °C) period. Values are given as mean ± standard deviation (n = 4 fillets).

	**dpm**	**C**	**ASC**	***Cc***	***Uo***	***Ap***	***Hp***	***p***
pH	1	6.67 ± 0.02 ^{A,a}	6.60 ± 0.02 $^{A, ab}$	6.58 ± 0.03 $^{A c}$	6.63 ± 0.02 $^{A, bd}$	6.57 ± 0.03 $^{A, c}$	6.56 ± 0.02 $^{A, c}$	0.042
	4	6.68 ± 0.04 ^{A}	6.65 ± 0.02 ^{B}	6.61 ± 0.02 ^{A}	6.65 ± 0.03 ^{A}	6.56 ± 0.02 ^{A}	6.58 ± 0.02 ^{A}	0.600
	6	6.70 ± 0.04 ^{A}	6.63 ± 0.02 ^{B}	6.59 ± 0.02 ^{A}	6.65 ± 0.03 ^{A}	6.59 ± 0.03 ^{A}	6.59 ± 0.04 ^{A}	0.297
	8	6.72 ± 0.02 $^{A, a}$	6.68 ± 0.03 $^{C, b}$	6.65 ± 0.03 $^{B, b}$	6.68 ± 0.02 $^{AB, b}$	6.61 ± 0.03 $^{A, c}$	6.66 ± 0.02 $^{B, b}$	0.001
	12	6.79 ± 0.02 $^{B, a}$	6.76 ± 0.04 $^{D, a}$	6.76 ± 0.05 $^{C, a}$	6.70 ± 0.03 $^{B, ab}$	6.66 ± 0.01 $^{B, b}$	6.72 ± 0.02 $^{C, ab}$	0.024
	p	0.045	<0.001	<0.001	0.002	0.045	<0.001	
WHC	1	71.0 ± 2.6 ^{C,a}	70.7 ± 2.1 ^{C,a}	70.8 ± 3.0 ^{B,a}	70.9 ± 1.3 ^{B,a}	71.2 ± 0.9 ^{B,a}	76.4 ± 0.9 ^{B,b}	<0.001
	4	70.6 ± 1.9 ^{C,a}	68.1 ± 2.3 ^{B,a}	70.1 ± 2.4 ^{B,a}	68.3 ± 1.5 ^{AB,a}	70.7 ± 1.4 ^{B,a}	76.5 ± 0.9 ^{B,b}	<0.001
	6	68.6 ± 1.8 ^{C}	68.5 ± 2.3 ^{B}	68.2 ± 1.4 ^{AB}	69.2 ± 1.3 ^{AB}	69.3 ± 0.8 ^{B}	71.4 ± 2.3 ^{A}	0.183
	8	66.6 ± 1.3 ^{B,a}	67.4 ± 1.6 ^{B,a}	67.5 ± 1.6 ^{AB,a}	68.3 ± 0.7 ^{AB,a}	68.1 ± 0.8 ^{A,a}	70.5 ± 0.6 ^{A,a}	0.004
	12	63.7 ± 1.9 ^{A,a}	64.9 ± 1.7 ^{A,ab}	66.2 ± 1.4 ^{A,b}	66.5 ± 1.4 ^{A,b}	66.3 ± 0.9 ^{A,b}	69.6 ± 1.1 ^{A,c}	<0.001
	p	<0.001	0.002	<0.001	<0.001	<0.001	0.002	

Superscript uppercase letters indicate differences attributable to storage time within each treatment. Superscript lowercase letters indicate differences attributable to treatments within each storage time ($p < 0.05$). dpm: days postmortem.
